# Determination of some selected secondary metabolites and their invitro antioxidant activity in commercially available Ethiopian tea (*Camellia sinensis*)

**DOI:** 10.1186/s40064-016-2056-1

**Published:** 2016-04-06

**Authors:** Dereje Bizuayehu, Minaleshewa Atlabachew, Mirtachew Tihar Ali

**Affiliations:** Department of Chemistry, Faculty of Science, Bahir Dar University, P.O. Box 79, Bahir Dar, Ethiopia

**Keywords:** Secondary metabolites, Tea, Polyphenol, Flavonoids, Tannins, Antioxidant activity

## Abstract

Eight brands of tea (*Camellia sinensis*),which are cultivated and commercially available in Ethiopian market, were analyzed for estimation of their total secondary metabolites (polyphenols, flavonoids and tannins) content and free radical scavenging activity which is expressed on dry weight basis. In this present study, the total polyphenols, tannin and flavonoid contents were studied spectrophotometrically using Folin–Dennis, Folin–Dennis/protein precipitation and aluminium chloride methods respectively. The free radical scavenging activity was determined by using DPPH radical assay. Results of the analysis revealed that the total polyphenol content varied from 21.3 ± 0.24 to 31.6 ± 0.31 mg of gallic acid equivalent/g of dry matter. Total flavonoids content in the tea samples varied from 8.17 ± 0.68 to 23.2 ± 0.68 mg of catechin equivalent/g of dry weight and tannin content varied from 5.64 ± 0.39 7.45 ± 0.27 mg tannic acid equivalent/g of dry weight basis. The free radical scavenging activity among the tea brand samples ranged from 28.8 ± 1.86 to 80.0 ± 0.63 mg ascorbic acid equivalent/g and the half maximal inhibitory concentration (IC_50%_) values varied from 7.3 ± 1.35 to 64.0 ± 2.81 µg/mL of extract. The correlation between the antioxidant activity with total polyphenol content (R = 0.91325), with flavonoids (R = 0.80658) and with tannin (R = 0.73125) was calculated and maximum correlation value was found between polyphenol content and the free radical scavenging activity of the tea samples. The results in this study also revealed that green tea had the higher polyphenolic content and found to have the most promising antioxidant activity. This study further confirmed that Ethiopia tea is reach in phenolic compounds as compared to some overseas tea cultivars/varieties.

## Background

Recent studies proven that substances from natural sources exhibits antioxidant properties that can be used to protect human beings from oxidative stress damage that could arise during physiological processes (Zeb 2015; Zeng et al. 2014). Tea, the leaves of the *C. sinensis* plant, is the most popular beverage consumed worldwide (Cabrera et al. 2006; Chaturvedula and Prakash 2011; Vasisht 2004). Tea consumption is mainly due to its desirable aroma, test and due to having various positive physiological functions (Hajimahmoodi et al. 2008).

Phytochemical investigations from tea leaves have demonstrated the presence of several constituents including flavonoids, phenolic compounds, alkaloids, tannins, volatile constituents and amino acids as main active ingredients having potent antioxidant activities, anti-carcinogenesis and hepatoprotective activities (Gramza et al. 2005; Kato and Shibamoto 2001; Pongsuwan et al. 2007; Vasisht 2004).

In the markets of world wide, three main types of tea (black tea, oolong tea, and green tea) are available based on the length of oxidation reaction and ways of processing. Of which, black tea is the most processed and has the greatest oxidation while Oolong tea is semi fermented or oxidized. On the other hand, green tea is obtained without oxidation but with little processing stage (Pongsuwan et al. 2007). As a result, type of tea processing has significant effect on the composition of the phyto constitutes and hence quality and bioactivity of the beverage (Kuroda and Hara 1999; Horie et al. 1993). Apart from the effect of processing on the composition and quality of tea leaves, the cultivar differences and environmental effects have meticulous contribution to the overall quality and composition of the metabolites. As a result, different varieties of green tea and processed tea are commercially available in the market recognized by the consumers.

Nowadays, the commercial tea in Ethiopia, which is black and green type, is processed from the *C. assamica* variety or the same type of *Camellia sinensis* L. grown in Wushwush, Gummero and Chewaka tea plantations located in Southwestern part of the country and more than ten different types of tea are commercially available for local and export market under different brand names originated from these plantations.

In the past, Ethiopia had little share from world market of tea. However, since the last one decade, the country has involved in exporting both green and black teas. Ethiopian tea is grown in the highland areas where dense forests are available; the soil is highly fertile and hence needs little or no fertilizers. As a result, Ethiopian tea has got popularity due its organic nature, aroma and natural flavor (http://www.ethiopianembassy.ie/trade/major-exports/tea-.html; http://www.southinvest.gov.et/Publications/SSNPR%20draft%20Profile/B/%20Black%20%20Green%20Tea%20Processing%20%20Packing.pdf).

Ethiopia has a unique climatic conditions and soil chemistry affecting the composition of plant secondary metabolites composition as it has been reflected in many studies on samples from Ethiopia. Our recent investigation on coffee, *Catha edulis* and potato samples grown in Ethiopia have indicated the dependence of phytochemicals composition of sample specimens on geographical origin (Atlabachew et al. 2015; Tadesse et al. 2015; Mehari et al. 2016).

Several reports have shown that the composition of total polyphenols in tea sample is one of the parameters for quality of tea regarding its biological properties, therefore, this assays should be applied for the quality control of manufactured teas (Fu et al. 2011). Thus, the determination of selected secondary metabolites (total polyphenols, total flavonoids and total tannins) content of tea is very important in assessing the standard and quality of tea as well as any potential implications to health. Since tea is a beverage which is a part of our daily dietary intake and frequently consumed, assessment of the nutrient composition especially the aforementioned ones and their antioxidant capacity in tea plant grown in Ethiopia is of great importance from quality and standards, nutrition and health perspectives.

A number of papers have been published regarding the determination of the metal content and other mineral of Ethiopian tea. However, to the best of our knowledge, no report has been made concerning the content of secondary metabolites such as polyphenols, in Ethiopian tea cultivars. Thus, the aim of this study was to evaluate total phenolic contents and antioxidant activities in tea crude extracts that are produced and commercially available in Ethiopia.

## Result and discussion

### Level of total polyphenols (TPC) in different tea brands

The content of total polyphenols in tea samples were derived from a standard curve of gallic acid ranging from 0.1 to 2.0 μg/mL (*y* = 0.01296*x* + 0.0093; R = 0.9913) and a standard curve of tannic acid ranging from 0.1 to 2.0 μg/mL (*y* = 0.00971*x* + 0.00845; R = 0.9957). The TPC was expressed as mg gallic acid equivalents per gram of sample (mg GAE/g) and mg tannic acid equivalent (mg TAE/g of sample) as shown in Table 1.

**Table 1:** Total polyphenols content of tea brands, the result was expressed in terms of GAE/g and TAE/g of sample for three independent measurements (triplicate; n = 3, mean ± SD)

Tea brands	TPC^A^ in mg GAE/g DW	TPC^B^ in mg TAE/g DW
Green tea	31. 6 ± 0.31^e^	42.1 ± 0.41^e^
Black lion	22.2 ± 0.39^b^	29.6 ± 0.52^b^
Eirmon	21.3 ± 0.24^a^	28.5 ± 0.31^a^
Gumero	25.0 ± 0.37^d^	32.3 ± 0.32^d^
Abyssinia	24.1 ± 0.78^c^	32.2 ± 0.04^c^
Dire	22.3 ± 0.50^b^	29.8 ± 0.66^b^
Addis	22.3 ± 0.50^b^	29.8 ± 0.66^b^
Wushwush	23.6 ± 0.54^c^	31.5 ± 0.72^c^

Values in the same column that are followed by a different letters (a–e) are significantly different p < 0.05 by Duncan’s multiple range tests

TPC^A^ is total phenol content, expressed in milligrams of gallic acid equivalents per g of dry tea sample

TPC^B^ is total phenol content, expressed in milligrams of tannic acid equivalents per g of dry tea sample

Looking at Table 1, the amount of total phenolic content varied with different tea brand and ranged from 21.2 ± 0.24 to 31. 6 ± 0.31 mg of GAE/g of DW or 28.5 ± 0.31 to 42.1 ± 0.41 mg of TAE/g of DW with maximum value in Green tea extract (31. 6 ± 0.31 mg GAE/g or 42.1 ± 0.41 mg of TAE/g) and minimum in Eirmon tea (21.3 ± 0.24 mg GAE/g DW or 28.5 mg TAE/g DW). The decreasing order of total polyphenols contents of tea crude extracts follow; Green tea > Gumero > Abbyssinia > Wushwush > Dire ≈ Addis > Black lion > Eirmon. Different letters in the Table 1 indicate the difference in TPC between the samples were significant (p < 0.05) when one way ANOVA Duncan’ multiple range test was carried out. The significant test result revealed that no significant difference (p > 0.05) was found between Abyssinia and Wushwush tea brands. The same trend was observed between Black lion, Addis and Dire tea brands.

The variation in polyphenol content in the studied commercial tea brands might be due to several factors such as age of the harvested tea plant, climate, agricultural practices, post harvest processing, and packaging. Black tea has found to contain less TPC than Green tea. Several reports from elsewhere have reported similar trend of phenolic composition between green and black tea. This is due to the fact that during fermentation process, some of the phenolic compounds undergo polymerization and/or degradation to other metabolites (Rusak et al. 2008; Ramamoorthy and Bono 2007).

Among the black tea brands, gumero brand had the highest (25.0 ± 0.37 mg GAE/g) while Eirmon had the lowest (21.3 ± 0.24 mg GAE/g) TPC as compared to the others. The difference in TPC within the black tea brands could be attributed to variation of factors like the agronomic conditions, harvested leaf age, and storage during and after transport, as well as the degree of fermentation.

According to Yoo et al. (2008) report on Korean tea, the total phenol content of green tea was found to be 7.69 mg GAE/100 g (7.69 mg GAE/g), while that of black tea was 745 mg GAE/100 g (7.45 mg GAE/g) (Yoo et al. 2008). Imran et al. (2013) examined the chemical profile of black tea in Pakistan with different solvent, the highest TPC, 1150 ± 15.0 mg GAE/100 g (11.5 ± 0.15 mg GAE/g) was recorded in ethanol followed by methanol (7.22 ± 0.12 mg GAE/g) while the lowest (3.54 ± 0.05 mg GAE/g) in water extract (Taheri and Sariri 2011). According to Nor Qhairul Izzreen and Mohd Fadzelly (2013), TPC and antioxidant activities of Malaysia tea leaves have shown variation with maturity level of the leaves. The authors have reported that the total polyphenols content was ranged from 56.6 ± 1.56 to 80.3 ± 0.61 mg GAE/g DW equivalent to Black tea (Mature) to green tea (Shoots) respectively. Ercisli et al. (2008) have reported the seasonal variation of total phenolic, antioxidant activity, plant nutritional elements, and fatty acids in tea leaves grown in Turkey. As per the report, the total phenolic content of tea leaves were 59.4 mg/g at 1st harvest and increased to 80.7 mg/g at 2nd harvest and decreased to 62.9 mg/g at 3rd harvest time (Ercisli et al. 2008). These results are significantly higher than the present data.

Thus, from the aforementioned reports, it is possible to conclude that variation in geographical origin have significant influence on the accumulation of phenolic compounds in tea in addition to other factors mentioned elsewhere.

### Level of total tannin in tea crude extracts

The content of total tannin was estimated by protein precipitation/binding method using egg albumin to precipitate tannins in the plant extracts (Atlabachew et al. 2014). This method is more realistic for estimating the content of tannins in plants because the method is closely related to biological effects of tannins (precipitation reaction of tannins with protein) due to the presence of several free phenolic hydroxyl groups that can form strong hydrogen bonds with proteins and carbohydrates (Peterson et al. 2005).

The concentration of total tannin in tea samples were derived from standard curve of tannic acid ranging from 0.1 to 2.0 μg/mL (*y* = 0.00971*x* + 0.00845; R = 0.9957). The results were determine by subtracting the total polyphenol content before treating with egg albumin (protein precipitation) (TPC_1_) minus the total polyphenols content after treating with egg albumin (protein precipitation) (TPC_2_) (Table 2).

**Table 2:** Total tannin content of tea brands, the results were expressed in terms of TAE/g the of sample for three independent measurements (triplicate; n = 3, mean ± SD)

Tea brands	TPC_1_^D^ mg TAE/g DW	TPC_2_^E^ mg/g DW	TTC^F^ mg TAE/g DW
Green tea	42.1 ± 0.41	34.6 ± 0.67	7.45 ± 0.23^d^
Black lion	29.6 ± 0.52	23.2 ± 1.05	6.42 ± 0.53^ab^
Eirmon	28.5 ± 0.31	21.8 ± 1.05	6.62 ± 0.77^bc^
Gumero	33.3 ± 0.32	26.5 ± 0.52	6.81 ± 0.21^bc^
Abyssinia	32.2 ± 1.04	25.5 ± 0.55	6.65 ± 0.84^bc^
Dire	29.8 ± 0.31	24.2 ± 0.17	5.64 ± 0.39^a^
Addis	29.8 ± 0.66	23.0 ± 0.67	6.90 ± 0.45^bc^
Wushwush	31.5 ± 0.72	25.5 ± 0.48	6.0 ± 0.25^ab^

Values in the same column that are followed by a different letters (a–d) are significantly different p < 0.05 by Duncan’s multiple range tests

TPC_1_^D^ is total polyphenol content expressed in terms of tannic acid equivalent per gram of dry weight before protein precipitation

TPC_2_^E^ is total polyphenol content expressed in terms of tannic acid equivalent per gram of dry weight after protein precipitation

TTC^F^ is total tannin content expressed in terms of tannic acid equivalent per gram of dry weight

The total tannin content among different tea brands was found to be 5.64 ± 0.39–7.45 ± 0.27 mg TAE/g DW corresponding to Dire tea to green tea brand. The one that has the ability to precipitate protein (egg albumin) efficiently will have high tannin content. Since protein precipitation is the unique characteristic feature of tannins (Peterson et al. 2005). Hence, the result revealed that green tea has the highest tannin content than other tea brands.

The comparative result of total tannin content of the studied tea brands can be also be depicted from Table 2. The order of the increment follows: Dire < Wushwush < Black lion < Abyssinia < Eirmon < Gumero < Addis < Green tea. The different letters on the graph indicated that the difference is significant (p < 0.05). So far only few papers are available in the literature. The tannin content of Indian tea which was determined by protein precipitation technique was 24 ± 2.8 mg TAE/g DW (Kumar 2011). And according to Bailey-Shaw et al., reported the total tannin content in commercially available black tea was ranged from 27.79 ± 0.20 to 68.13 ± 1.25 mg TAE/g DW (Bailey-Shaw et al. 2012).

### The level of total flavonoid in tea crude extracts

The total flavonoid content in tea samples were estimated from a standard curve of catechin ranging from 0.10 to 3.00 μg/mL (*y* = 0.00439*x* − 0.000974: R = 0.9985).TFC was expressed as mg catechin equivalents (CE)/g DW). The measurements were carried out three times (triplicate) for each tea extract and results were reported in (mean ± SD, n = 3) Table 3.

**Table 3:** Total flavonoids content (TFC) of tea brands, the results were expressed in terms of CE/g the of sample for three independent measurements (triplicate; n = 3, mean ± SD)

Tea brands	TFC^C^ in mg CE/g dry weight
Green tea	23.2 ± 0.68^d^
Black lion	11.1 ± 1.25^b^
Eirmon	14.7 ± 1.25^c^
Gumero	10.1 ± 0.95^b^
Abyssinia	13.9 ± 0.35^c^
Dire	8.17 ± 0.67^a^
Addis	11.6 ± 0.10^b^
Wushwush	11.1 ± 0.68^b^

Values in the same column that are followed by a different letters (a–d) are significantly different p < 0.05 by Duncan’s multiple range tests

TFC^C^ is total flavonoid content, expressed as mg catechin equivalent per gram of dried sample

According to the result shown in the Table 3, the total flavonoid content in the studied tea brands ranged from 8.17 ± 0.68 (Dire tea) to 23.2 ± 0.68 mg CE/g DW (Green tea). Hence, from the results presented, Green tea had the highest total flavonoid content (23.2 ± 0.68 mg CE/g DW), where as Dire tea showed the lowest total flavonoid content (8.17 ± 0.68 mg CE/g DW) among the tested tea brands. The most possible reason that led to vary the flavonoid content among tea samples might be due to degree of fermentation. Since green tea is prepared without fermentation, rather the freshly harvested leaves are steamed immediately to inactivate the enzymes polyphenol oxidase to prevent oxidation and polymerization of primary polyphenols, as result most polyphenols remain intact (Kris-Etherton and Keen 2002). In black tea, during the fermentation process most of the polyphenols especially catechins, forming the main flavanols of green tea, are either oxidized and polymerized to theaflavins (TF) and thearubigins (TR) or degraded to other form (Khomdram and Singh 2011). As a result the total flavonoid content of green tea is expectedly higher than black tea brands.

According to Nor Qhairul Izzreen and Mohd Fadzelly (2013), the total flavonoids content of Malaysian tea was ranged from 19.07 ± 1.46 to 35.17 ± 0.91 mg QE/g DW equivalent to black tea (Mature) to green tea (shoots).These results are comparable to the current findings. Comparable results were also reported on Chinese green and black tea (Hajimahmoodi et al. 2008). However, Korean green and black teas have contained 4.126 mg CE/g) and (4.31 mg CE/g) respectively (Yoo et al. 2008). These results are by far lower than our findings which signify the dependence of these metabolites on geographical origin and other factors.

By comparing the content of total flavonoids in the studied samples, the following trend was arranged in an ascending order: Dire < Gumero < Black lion < Wushwush < Addis < Abyssinia < Eirmon < Green tea (Table 3). Different letters that indicated within a column of Table 3 showed the presence of significant different in total flavonoid (TFC) content among tea brands at 95 % confidence interval. Thus, Eirmon, Abyssinia, Addis and Wushwush tea brands hadn’t any significance difference in TFC. The same is true for Black lion and Gumero tea brands at (p < 0.05).

### Comparison of total polyphenol, flavonoid and tannin content among tea brands

**Fig. 1:**
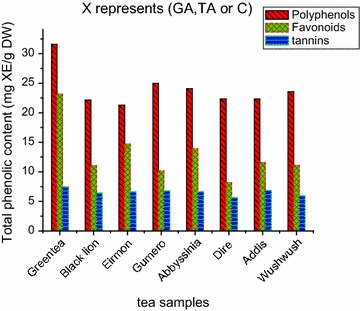
The level of total polyphenols, flavonoid and tannin content of different tea brands

The highest total polyphenols, flavonoid, and tannin contents were observed in green tea crude extract; however, the black tea brands showed variation in the content of these bioactive molecules. For example gumero tea has the highest TPC but the second lowest in TFC. In the case of TTC Addis tea extract showed the highest while Dire tea extract has the lowest among the studied black tea brands. The results also revealed that Gumero and Dire tea brands had the first and the last rank among the black tea brands in TPC and TTC respectively (Fig. 1).

### The percentage inhibition of DPPH with tea crude extract

The free radical scavenging activity (percentage of inhibition) of tea brands with different volume of tea crude extracts (75, 50, 25 and 10 µL) was evaluated. The percentage inhibition that found with respect to each volume of extract was used to plot a graph, based on the % inhibition *Vs* concentration of sample in (µg/mL). From graph the concentration of the tea extracts leading to 50 % reduction of the initial DPPH concentration, (IC_50%_) was estimated (Table 4) and the antioxidant activity of the tea sample relative to ascorbic acid equivalent per gram of sample was evaluated from the plot of percentage inhibition of ascorbic acid *Vs* amount of ascorbic acid in µg.

**Table 4:** The antioxidant activity of tea brands in mg of AAE/g DW and IC_50_ values

Tea samples	AOA^G^ mg AAE/g DW	IC_50%_ value (µg/mL)
Green tea	80.0 ± 0.63^e^	7.303 ± 1.35^a^
Black lion	28.8 ± 1.86^a^	56.067 ± 2.02^c^
Eirmon	41.9 ± 0.87^c^	51.320 ± 1.45^b^
Gumero	52.3 ± 2.91^d^	47.086 ± 2.04^b^
Abyssinia	42.9 ± 2.07^bc^	51.325 ± 1.69^b^
Dire	40.7 ± 2.72^bc^	64.015 ± 2.81^d^
Addis	40.8 ± 3.40b^c^	51.145 ± 2.85^b^
Wushwush	32.6 ± 1.92^a^	61.830 ± 2.37^d^

Values in the same column that are followed by a different letters (a–e) are significantly different p < 0.05 by Duncan’s multiple range tests

AOA^G^ is the antioxidant activity, expressed in milligrams of ascorbic equivalents per g of dry tea sample

### The antioxidant activity of crude extracts of different tea brands

The antioxidant activity of tea samples were estimated from the regression equation of ascorbic acid standard ranging from 0.25 to 1.825 µg/mL and the curve was % inhibition *Vs* amount of ascorbic acid in µg (*y* = 11.16862*x* + 0.02734, R = 0.998). Where, y is percentage inhibition, and x is amount of ascorbic acid (µg). The percentage inhibition for each of the extract were reported as mg of AAE/g of dried sample for triplicate measurement (n = 3, mean ± SD) Table 4.

Comparing with literature values, the present result (Table 4) are comparable with some of the reports (Hajimahmoodi et al. 2008), while wide variations were seen when compared with samples from elsewhere as it was observed for TPC, TFC and TTCs (Yoo et al. 2008).

### The IC_50_ (EC_50_) values of each type of tea brand

The efficient concentration (EC_50_) or half maximal inhibitory concentration (IC_50_) value, defined as the concentration of the sample leading to 50 % reduction of the initial DPPH concentration, was calculated from the linear regression of plots of percentage inhibition (% DPPH scavenging activity) against concentration of tea extracts (y = bx + c) where, y is the percentage of inhibition for each of tea extract, x is concentration (µg/mL) and b is the y intercept. The values of IC_50%_ were expressed in µg/mL and presented in Table 4.

The IC_50%_ values of tea extracts were ranged from 7.30 to 64.0 μg/mL, which equivalent to green tea extract to Dire tea extract. This implies that the concentration of green tea sample required decreasing the initial concentration of DPPH solution by 50 % is 7.303 μg/ml whereas Dire tea is 64.1 μg/ml. The result showed that IC_50%_ value is negatively related to the antioxidant activity, as it expresses the amount of antioxidant needed to decrease its radical concentration by 50 %. The lower the IC_50%_ value, the higher is the antioxidant activity of the test sample. Therefore, those tea extracts having the minimum amount of IC_50%_ values could have high antioxidant activity DPPH free radical scavenging.

### The correlation between the total phenolic compounds and the antioxidant activity

**Fig. 2:**
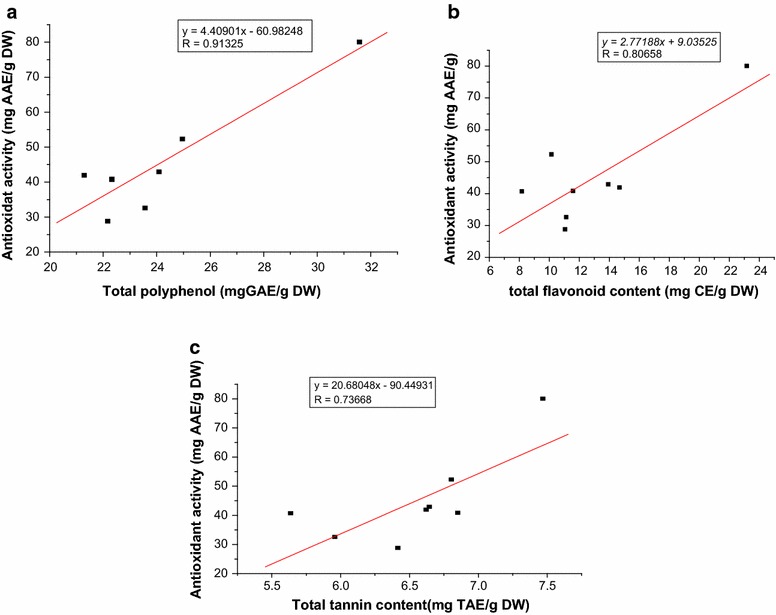
The correlation between the total phenolic (**a**), total flavonoids (**b**) and total tannins (**c**) content with the antioxidant activity

The relation between antioxidant activities and the total phenolic content of the eight tea crude extracts was examined by plotting antioxidant activity (Y) *Vs* total phenolic content (X) as shown in Fig. 2a–c. By comparing the correlation coefficients (R values) one can identify which phenolic groups are highly responsible for antioxidant activity.

The level of total polyphenols, flavonoids and tannins contents have been found to positively correlate with the antioxidant activity in the tea crude extracts. The result showed that a positive linear correlation between the antioxidant capacities and total polyphenols (R = 0.91325), flavonoids (R = 0.80568) and tannins (R = 0.73125) content respectively. However total polyphenols content was strongly correlated (R = 0.91325) with antioxidant activity as compare to flavonoids and tannins, which indicated that polyphenols could be one of the main components responsible for antioxidant activities of these beverages. Some studies show that there is strong relationship between antioxidant activity and total polyphenols content in tea leave extract (Turkmen et al. 2007).

## Conclusions

The present study was undertaken to estimate the total polyphenol, flavonoid, tannin contents and their in-vitro antioxidant activity in eight brands of Ethiopian tea. The result demonstrated that total phenolic content and antioxidative potential of non fermented tea (green tea) is greater than fermented tea (black tea) in all parameters under consideration. A positive relationship between antioxidant activities and total phenolic contents was also observed. However, antioxidant activity and total polyphenol content was well correlated as compare to other parameters. This leads to conclude polyphenols are the main components of tea, which are responsible for antioxidant activities of tea beverages. Although all the evidence from research on green tea is very promising, most people in Ethiopia aren’t familiar with its medicinal value. Therefore, future studies are necessary to fully understand its contributions to human health, and recommend its regular consumption in diets, in which green tea consumption is nowadays limited and sporadic in Ethiopia. Ethiopian teas have found to contain significantly higher concentration of these secondary metabolites as compared to tea samples reported from overseas.

## Methods

### Instrument and apparatus

Analytical digital balance (Ohaus, Swizerland), Magnetic stirrer, Centrifuge (CENTURION SCIENTIFIC LTD, UK), Separatory funnel, Whatman filter paper, PH meter and UV–Visible spectrometer (SANYO SP65 UV/Vis, UK) equipped with 1 cm cuvette were used.

### Chemicals and reagents

For the determination of polyphenolic compounds and their antioxidant activity in tea samples, the following chemicals and reagents were used; 2,2-diphenyl-1-picrylhydrazyl DPPH and d-Catechin, (Sigma Alderich), Egg albumin, Ethanol (CH_3_CH_2_OH), Glacial acetic acid (CH_3_COOH), Anhydrous aluminium chloride (AlCl_3_), Sodium acetate (CH_3_COONa), Sodium chloride(NaCl), Anhydrous sodium nitrite (NaNO_2_), Sodium hydroxide (NaOH), Sodium carbonate (Na_2_CO_3_) (BLULUX Laboratories (p) Ltd, India). O-phosphoric acid, H_3_PO_4_ (Fisher Scientific Limited, UK), Phosphomolybdic acid ((H_3_P (Mo_13_O_14_)_4_·H_2_O) and Sodium tungstate hydrated (Na_2_WO_4_·2H_2_O) (Scharlau Chememia), Ascorbic acid and (BDH, England) Gallic acid powder (C_7_H_6_O_5_). All chemicals were analytical grade chemicals. Distilled water was used throughout the experiment for rinsing as well as dilution purpose.

### Sample collection

Detailed description of the tea brands and the amount of samples collected from each brand are summarized in Table 5.

**Table 5:** Description of sample brands

No.	Tea brand name	Tea form	Amount per box	Country origin	Country packed in	Area grown	Sample collected, box
1	Green tea	Leaf	25 bags	Ethiopia	Ethiopia	WTP and GTP	5
2	Addis	Leaf	25 bags	Ethiopia	Ethiopia	WTP and GTP	5
3	Black lion	Leaf	80 g	Ethiopia	Ethiopia	CTP	5
4	Eirmon	Leaf	50 g	Ethiopia	Ethiopia	WTP and GTP	5
5	Wushwush	Leaf	100 g	Ethiopia	Ethiopia	WTP	5
6	Gumero	Leaf	100 g	Ethiopia	Ethiopia	GTP	5
7	Dire	Leaf	50 g	Ethiopia	Ethiopia	WTP	5
8	Abyssinia	Leaf	–	Ethiopia	Ethiopia	WTP and GTP	12 bags

*WTP* Wushwush tea plantation, *GTP* Gumero tea plantation, *CTP* Chewaka tea plantation

### Sample preparation

Prior to extraction, each type of tea sample was ground using stainless steel grinder to obtain a homogeneous fine powder. The extraction was carried out by transferring 0.5 g of each ground tea sample into 25 mL separate beakers and 10 mL of ethanol–water mixture (80:20) was added into beakers. Then, the extraction was carried out for 2 hours with continuous mixing by magnetic stirrer at room temperature. The turbid solutions were poured into plastic test tubes and centrifuged at 2000 rpm for 20 min, then filtered through Whatman paper filters into 100 mL volumetric flask. The pellets/residues were re-extracted with the same condition and pooled together the extracts, then diluted to the volume appropriately with the extracting solvent according to each specific assay. The resulting crude extracts were used for the estimation of total polyphenols, flavonoids, and tannins as well as their in vitro antioxidant activities.

### Analysis of secondary metabolites

#### Determination of total polyphenol content

The Folin–Denis method reported by Hajimahmoodi et al. (2008) with slight modification was used in this study for the estimation of total polyphenol content in the tea brands. Aliquots of 100 µL tea sample extracts were placed into the respective 10 mL test tubes followed by 1 mL of Folin–Denis reagent. At the end of this period 1 mL of 7 % (w/v) sodium carbonate (Na_2_CO_3_) solution was added and 10 more minutes were allowed. The volume was adjusted to 10 mL (analyzed solution) and mixed thoroughly and then the test tubes were kept at room temperature for 1 h. Absorbance was measured at 760 nm. The results were expressed as mg gallic acid equivalent per g of dry sample (mg GAE/g). The determination was carried out for three independently prepared samples of each type of tea brands. The blanks were prepared with the same procedure. Reagent blank was prepared from (1 mL Folin–Denis + 1 mL 7 % Na_2_CO_3_ + solvent) and sample blank (100 µL crude extract + 1 mL 7 % Na_2_CO_3_ + solvent). These are termed as double blank, the aim was to minimize the potential interference that comes from during reagent/standard preparation as well as sample preparation. To achieve this in all absorbance measurement double blank subtraction was carried out for each assay.

#### Determination of total tannins

The content of total tannin was determined by protein precipitation/binding method using the method reported by Atlabachew et al. (2014) using egg albumin to precipitate tannins in the plant extracts. A portion of 0.3 mL the sample extract was properly diluted to 2 mL with distilled water and mixed with 2 mL of 5 mg/mL egg albumin in acetate buffer solution (0.1 M, pH, 4.6), which was previously prepared, and kept for 15 min, centrifuged at 2000 rpm for 20 min and supernatant was filtered with filter paper and used for analysis. Exactly 400 µL of the supernatant was taken and 1 mL of Folin–Denis reagent was added, followed by 1 mL of 7 % (w/v) sodium carbonate solution (Na_2_CO_3_), and then filled with distilled water to 10 mL. The light blue colored solution was measured using UV/Vis spectrophotometer at 760 nm. For tannin determination, first total polyphenols in the tea extracts before protein precipitation was determined using Folin–Ciocalteu reagent (TPC_1_). Then the same volume of the extract was treated with protein (egg albumin) and kept for some time. Then, after a set period of time, the solution was centrifuged and the precipitated was kept for the second, the supernatant (which is free from tannins) was taken and the concentration of non-tannin phenolic compounds was determined using the above procedure. Then, tannin concentration was determined by difference. Calibration curve was constructed using tannic acid in ethanol–water mixture; the result was expressed with tannic acid equivalent per dry weight of the sample.

#### Determination of total flavonoids

Total flavonoid content was measured by making use of aluminum chloride colorimetric assay method reported by Yoo et al. (2008). An aliquot (200 µL) of extract was added to 10 mL test tube containing 5 mL of distilled water. To the tubes 250 µL of 5 % NaNO_2_ was added, after 5 min, 500 µL of 10 % AlCl_3_ was added followed by 2 mL of 1 M NaOH at the 6th min. The total volume was made up to 10 mL with distilled water (analyzed sample solution). The solution was mixed well and absorbance of the mixture, pink in colour was measured against prepared reagent blank at 510 nm using SP65 UV/Vis spectrophotometer. Total flavonoid content was expressed as mg catechin equivalents per gram of dry mass of tea (mg CE/g). Determination was made in triplicate. Double blank was prepared; (1) sample blank was prepared from 0.2 mL of tea extract + 0.25 mL of 5 % NaNO_2_ + 2 mL of 1 M NaOH + the rest distilled water. And reagent blank was prepared from 0.5 mL 10 % AlCl_3_ + 0.25 % NaNO_2_ + 2 mL NaOH + 7.25 mL solvent.

#### Measuring of in vitro antioxidant activity

The antioxidant activity of the tea extracts were evaluated using DPPH method according to the method reported by Rohman et al. (2010) but with little modification. From previously prepared diluted sample extracts different concentrations/volumes (75, 50, 25 and 10 µL) of each extract was poured into four separate test tubes. Then, to each of the extract 1 mL of 0.1 mM DPPH solution was added. The volume was adjusted to 4 mL with the solvent. The mixture was vigorously shaken, homogenized, left for 30 min, and then the absorbance was read a 517 nm. For each samples three independent measurements were carried out. Ascorbic acid with different concentration was used for constructing calibration curve.

### Statistical analysis

Each parameter which is under consideration was carried out three times from which the mean values and their respective standard deviation were calculated. Significant differences of the data among the parameters were calculated by performing one way analysis of variance (ANOVA) test using SPSS vesrsion20 software. Sample means were compared by least significant difference (LSD) multiple Duncan’s range test. Differences at (p < 0.05) were considered to be significant. Correlation analyses of free radical scavenging (antioxidant) activity (Y) versus the total phenolic content, flavonoids and tannin (X) were also carried out by using origin 6.1 software.
